# Exceptional Prices of Medical and Other Supplies during the COVID-19 Pandemic in Ecuador

**DOI:** 10.4269/ajtmh.21-0221

**Published:** 2021-05-20

**Authors:** Esteban Ortiz-Prado, Raul Fernandez-Naranjo, Yeferson Torres-Berru, Rachel Lowe, Irene Torres

**Affiliations:** 1 One Health Research Group, Faculty of Medicine, Universidad de las Americas, Quito, Ecuador;; 2 Department of Cell Biology, Physiology and Immunology, Universidad de Barcelona, Barcelona, Spain;; 3 University of Salamanca, Salamanca, Spain;; 4 Instituto Superior Tecnológico Sudamericano, Loja, Ecuador;; 5 Centre for Mathematical Modelling of Infectious Diseases, London School of Hygiene & Tropical Medicine, London, United Kingdom;; 6 Centre on Climate Change and Planetary Health, London School of Hygiene & Tropical Medicine, London, United Kingdom;; 7 Fundacion Octaedro, Quito, Ecuador

## Abstract

Shortages of essential supplies used to prevent, diagnose, and treat COVID-19 have been a global concern, and price speculation and hikes may have negatively influenced access. This study identifies variability in prices of products acquired through government-driven contracts in Ecuador during the early pandemic response, when the highest mortality rates were registered in a single day. Data were obtained from the National Public Procurement Service (SERCOP) database between March 1 and July 31, 2020. A statistical descriptive analysis was conducted to extract relevant measures for commonly purchased products, medical devices, pharmaceutical drugs, and other goods. Among the most frequently purchased products, the greatest amounts were spent on face masks (US$4.5 million), acetaminophen (US$2.2 million), and reverse transcriptase quantitative polymerase chain reaction assay kits (US$1.8 million). Prices varied greatly, depending on each individual contract and on the number of units purchased; some were exceptionally higher than their market value. Compared with 2019, the mean price of medical examination gloves increased up to 1,307%, acetaminophen 500 mg pills, up to 796%, and oxygen flasks, 30.8%. In a context of budgetary constraints that actually required an effective use of available funds, speculative price hikes may have limited patient access to health care and the protection of the general population and health care workers. COVID-19 vaccine allocations to privileged individuals have also been widely reported. Price caps and other forms of regulation, as well as greater scrutiny and transparency of government-driven purchases, and investment in local production, are warranted in Ecuador for improved infectious disease prevention.

## INTRODUCTION

Shortages of essential supplies used to prevent, diagnose, and treat COVID-19 have been documented around the world, with the pandemic exacerbating the impact of systemic weaknesses on availability in places with high transmission rates.^1^ Lack of medical devices such as mechanical ventilators, reverse transcriptase quantitative polymerase chain reaction (RT-qPCR) test kits, and a full range of personal protection equipment (PPE) for health care workers to safely comply with clinical duty has not been the only cause for concern; limited access to basic food supplies may increase exposure and vulnerability to the disease.^2^

Breaches of anticorruption standards, including surcharges, speculation, collusion, cutting corners in procurement processes, and even politicians taking advantage of the crisis to increase their private benefits, have been recorded in various countries during the COVID-19 pandemic.^3^ Government transparency in decision-making has been described as a major problem in response efforts worldwide,^4^ together with weak regulations and accountability mechanisms and low wages in the heath sector.^5^ In countries such as the United Kingdom, a National Health Service official was found selling PPE on the side, and the U.S. administration paid extraordinary prices to third-party vendors.^6^

Ecuador is a South American country of 17 million people, which was severely hit early in the pandemic, registering more than 40,000 deaths in 2020, one of the highest rates per capita in the world. Although the government declared a health emergency in March followed by a national lockdown, social protection measures and health system resources have been limited. Vulnerable populations have also relied, for example, on food kits from the World Food Program^7^ and provisionally lower-priced food and drug outlets supported by the peasant movement (Movimiento Social Campesino, FECAOL, in Spanish).^8,9^

Most medical equipment, devices, and medicines are imported into Ecuador, and the public health sector’s resource allocation has forced populations to pay out of pocket for medication in the past, placing those who cannot pay at greater risk.^10^ As the country’s health system became overstretched to the point of collapse in Guayas, the first COVID-19 hotspot,^11,12^ major corruption scandals emerged in relation to the procurement of medical devices such as face masks or human remains pouches (HRPs), commonly known as body bags.^13^ Even though the law requires civil society organizations’ participation in Emergency Operations Committee meetings, they have not been present during the sessions convened for the pandemic response, which has limited transparency in decision-making.^14^ In addition, as Ecuador was seeing a steady rise in the number of confirmed cases early in the pandemic, its diagnostic capacity did not increase to meet demands.^15^

Ecuador mandated face masks on April 7, 2020, for the general population,^16^ but these were not freely distributed by the government nor was a price cap established. In practice, this implied that only people with enough means could purchase them to remain protected.^17^ Similarly, in countries with limited resources, those who can afford expensive private testing may presumably be at an advantage over those who cannot. With only 14% of medicines being produced locally, and 86% of this revenue going to a single company,^18^ market prices are not necessarily competitive. It has been reported that at least 202 public contracts signed during the pandemic response have irregularities; a majority of them involve the purchase of medical supplies, masks, suits, antibacterial gel, PCR tests, medicines, food kits, and other products and services.^19^ The comptroller general of Ecuador found that food kits were purchased by public institutions for distribution among vulnerable groups across the country at a much higher cost than regular store prices.^20,21^ Furthermore, tocilizumab vials donated by a pharmaceutical company to the government were sold on the black market for at least 5 and up to 10 times their market price in April 2020.^22^ Finally, oxygen shortages and exceedingly high prices were also reported by the media in early April of the same year.^23^

This study aims to compare variability in prices of products acquired through public contracts in Ecuador for use in clinical settings or as protection for the general population in response to the COVID-19 pandemic.

## METHODS

### Study design.

This is an observational descriptive study of the publicly available data on government-driven purchases for the public health system in Ecuador from January 1, 2020 to July 31, 2020.

### Setting.

The study was conducted in Ecuador, a country located in South America, with an estimated population of 17.68 million. The National Public Procurement Service (SERCOP) is in charge of promoting citizen participation, increasing access to and use of public information by the population, increasing transparency, and fighting fraud and corruption that could arise from bad practices in public procurement. Our study quantifies differences in prices of commonly purchased devices, medicines, and supplies in public contracts registered in SERCOP that have been identified as relating to the COVID-19 pandemic.

### Variables.

Considering the Uniform Resource Locator (URL) of each contracting process as input, the following sections were identified: description, dates, products, qualification parameters, invitations, files, and questions from suppliers. Each section was extracted through scraping according to its equivalent identification in html and was stored in an unrelated database. The variables analyzed were as follows: governmental institution responsible for the purchase process (Ministry, Central Government, Decentralized Autonomous Governments), location where the contract was based (Municipality and Province), type of product or service, unit price, and type of contract (direct contract or public auction).

### Data sources.

Data were obtained from the publicly available database from SERCOP. Data on government-driven purchase processes between March 1 and July 31, 2020, were extracted using Selenium with Python. Using fuzzy matching and regular expressions, we identified the most frequent products in the bids and contracts. The source code of python script is available in the following link: https://github.com/torresyeferson/DattaMinigCorruption/blob/main/Scraping.py

### Statistical method.

A statistical descriptive analysis was conducted to extract relevant measures for each product and determine variability across all items. Tableau software was used for visualization and analysis. A descriptive statistical analysis was performed to describe means, trends and percentage changes.

## RESULTS

### Data by institution.

The highest spenders during the peak of the pandemic in 2020 were the Guayaquil Municipal Government (US$19 million) and the Guayas Provincial Government (US$5 million), followed by the Ministry of Public Health (US$18 million). Hospitals that spent the largest sums were also located in Guayaquil—Sagrado Corazon de Jesus and Abel Gilbert Ponton—which spent approximately US$5 million each (Table 1).

**Table 1:** Expenditures of local hospitals (March 1–May 31, 2020)

Hospital	March	April	May	Total	% Of total
	(in US$)	(in US$)	(in US$)	(in US$)	
Hospital Sagrado Corazon de Jesus	2,845,134	2,519,196	–	5,364,330	14%
Hospital Abel Gilbert Ponton	4,581,303	249,207	–	4,830,510	13%
Hospital Quito-Sur	2,799,047	–	–	2,799,047	7%
Hospital Martin Icaza	1,555,057	705,002	153,566	2,413,625	6%
Hospital General de Machala	1,669,934	391,820	–	2,061,754	5%
Hospital General Enrique Garcés	1,965,252	–	–	1,965,252	5%
Hospital de Especialidades Carlos Andrade Marin	1,826,454	58,500	–	1,884,954	5%
Hospital del Niño	1,840,205	24,754	–	1,864,959	5%
Hospital General Monte Sinaí	1,421,120	426,689	–	1,847,809	5%
Hospital General de Milagro	1,529,479	188,787	–	1,718,266	5%
Hospital General Guasmo Sur	1,666,462	–	–	1,666,462	4%
Hospital de Especialidades Eugenio Espejo	1,600,469	–	–	1,600,469	4%
Hospital de Especialidades Fuerzas Armadas No. 1	557,525	682,018	117,467	1,357,010	4%
Hospital General Marco Vinicio Iza	588,664	656,694	–	1,245,358	3%
Hospital General de Manta	1,196,829	45,298	–	1,242,127	3%
Hospital de Especialidades Jose Carrasco Arteaga	989,979	220,553	–	1,210,532	3%
Hospital Vicente Corral Moscoso	979,148	45,269	–	1,024,417	3%
Hospital Rodríguez Zambrano	963,248	–	–	963,248	3%
Hospital General de Ambato	848,625	–	–	848,625	2%
Total	31,423,934	6,213,787	271,033	37,908,754	100%

As a reference, Ecuador has 168 public hospitals; its 19 highest spenders used 1.4% of Ecuador’s 2018 total public health budget (US$2.665 million) in only 3 months, between March 1 and May 31, 2020.^24^

### Data by product or service.

The most frequently purchased products in public contracts were medical devices (ventilators), laboratory supplies (RT-qPCR assay kits), all types of facial masks (including N95 respirators, surgical masks, and face masks), medicines (acetaminophen, piperacillin, hydroxychloroquine), disinfectant (quaternary ammonium), HRPs, and food kits.

### Data by prices.

**Figure 1.**
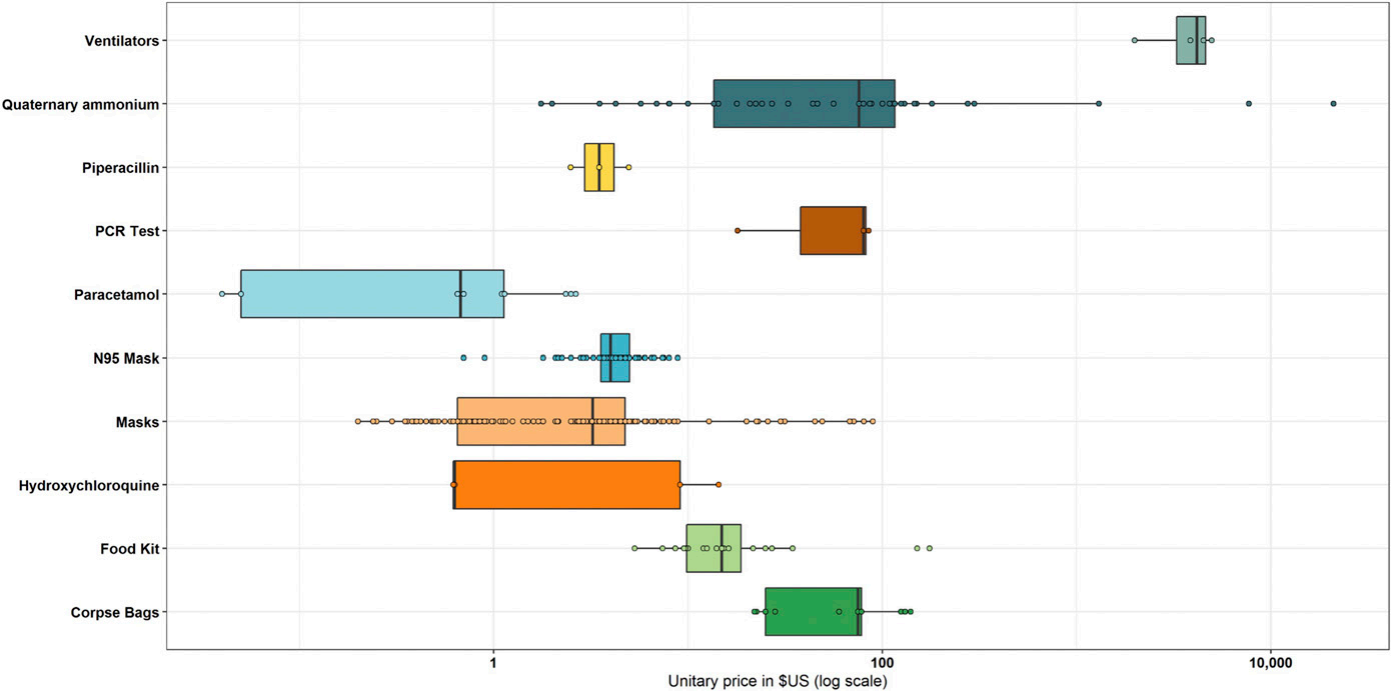
Price distribution of basic supplies related to the COVID-19 response in Ecuador (March 1–July 31, 2020). The plot depicts variations in price by item compared with the median/mean price, using as reference outliers in distribution in a logarithmic scale. Dots outside bars and boxes represent extreme and atypical values.

Prices varied by contract and depended on the number of units purchased. Distribution of prices for all main products are included in Figure 1. During the study period, prices were analyzed by item, institution, and month, ranging from individual products as cheap as US$0.03 (acetaminophen 500 mg pills) to US$140,000 (mechanical ventilators). A total of US$4.5 million was spent on all types of masks (including N95 respirators, surgical masks, and face masks), ranging from US$0.50 to US$90 per unit. The government spent at least US$2.2 million on acetaminophen, with prices ranging from US$0.04 per 500-mg pill to US$2.65 per oral suspension bottle (different sizes). In terms of diagnostic supplies, at least US$1.8 million were spent on RT-qPCR essay kits. The lowest price for an individual primer was US$18 (per reaction) and the highest was US$85 per reaction when including the extraction kit. The price of an individual HRP reached exceptional high values per unit. For instance, the Ecuadorian Institute of Social Security (IESS) bought 4,000 units at US$148.5 per HRP, a value 493% higher than the market price of US$25.^13^ Prices of individual items per unit can be found in the link shared in the Availability of Data section.

**Figure 2.**
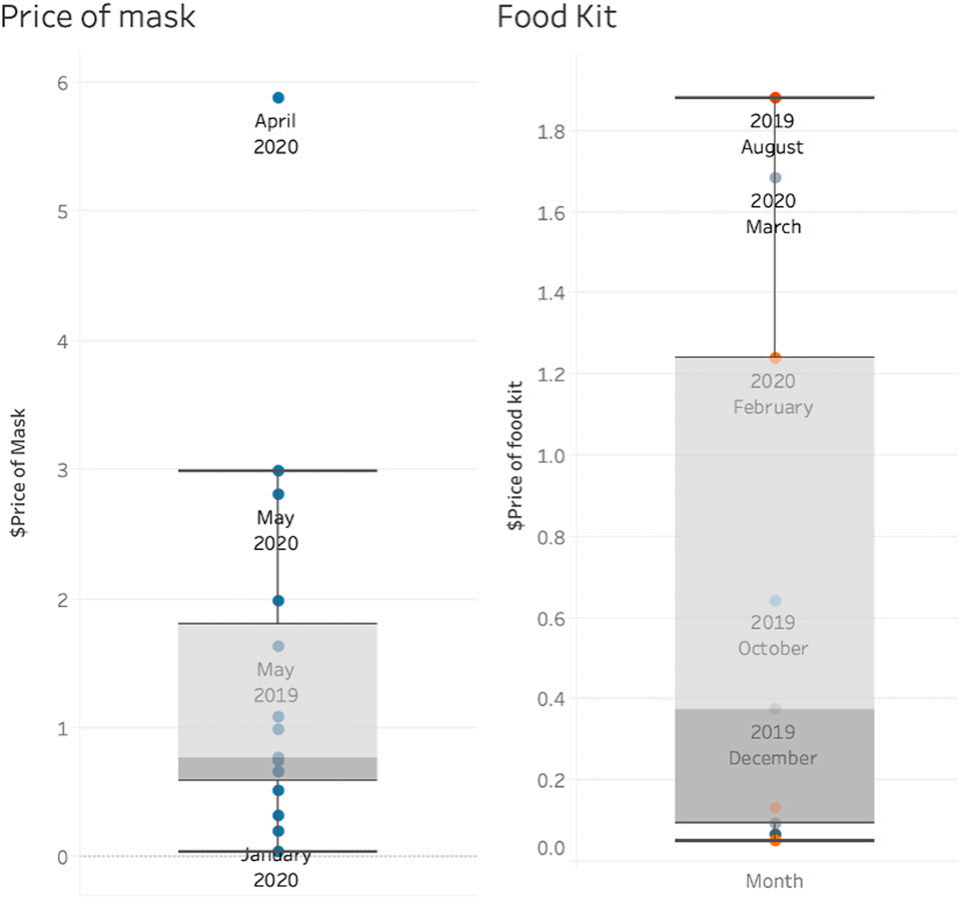
Price distribution of face masks and food kits in Ecuador by month (August 1, 2019–May 31, 2020). We selected these two items for comparison because contracts most frequently included them; data for other items were not available for the 2 years. This figure appears in color at www.ajtmh.org.

In another exceptional purchase, the National Secretariat for Risk Management acquired 7,000 food kits for vulnerable families, at US$150.82 per hamper. The Comptroller General of Ecuador has subsequently established that the market value of each food kit is US$95.16, one of the reasons being that only eight of the 18 products are taxable, but the vendor had included a tax value for all items.^21^ Furthermore, the report indicates prices were also inflated.

### Data by dates.

**Figure 3.**
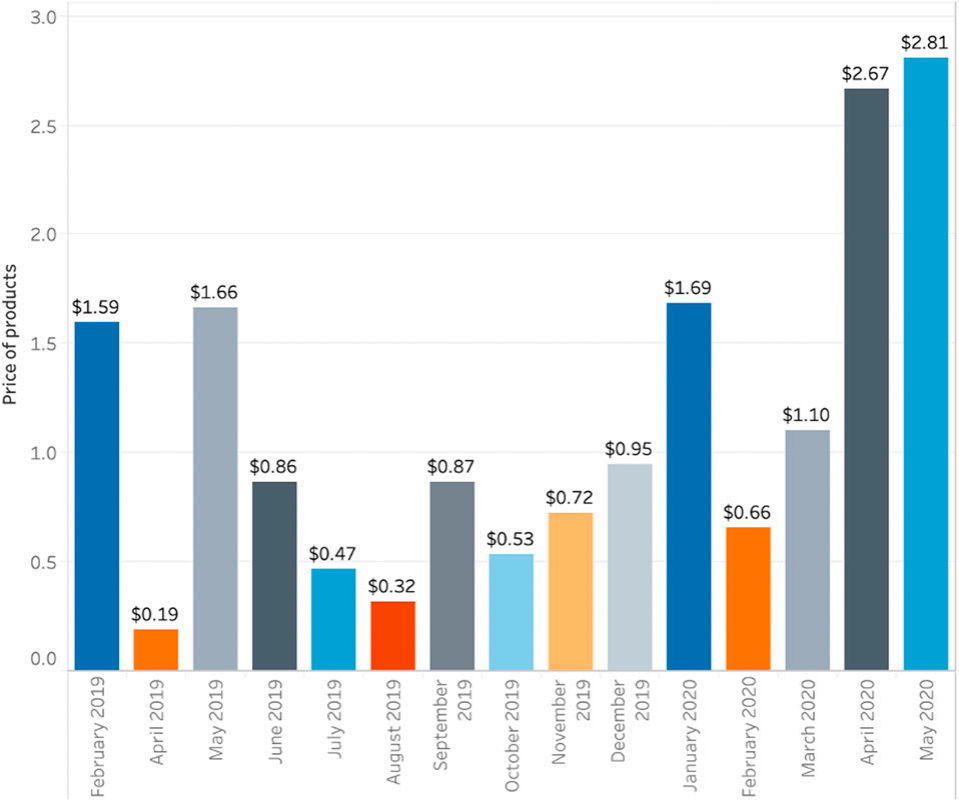
Monthly price of face masks in public contracts (there was no information for January and March 2019). This figure appears in color at www.ajtmh.org.

The prices of food kits and medical supplies such as face masks experienced sharp increases during the first few months of the pandemic (Figure 2). Although the prices of face masks oscillated greatly since at least 2019, the increase in April and May 2020 is evident (Figure 3).

Most of the increase was on products that were highly needed during the health emergency caused by the COVID-19 pandemic. Compared with the previous year, medical exam gloves increased by 1,307%, acetaminophen 500-mg pills by 796%, and oxygen flasks (different sizes) by 30.8% in the corresponding month during which data were available (Table 2).

**Table 2:** Comparison of mean prices between January 1, 2019 and May 30, 2020*

	Month	2019	2020	% Increase
Medical exam and sterile gloves (100 units)	Jan	$0.03	$0.07	144%
	April	$0.19	$2.67	1,307%
	May	$1.66	$2.81	69%
Acetaminophen (500-mg pill)	Jan	$0.002	$0.014	796%
	April	$0.002	$0.004	166%
Oxygen (flasks, different sizes)	April	$2.82	$3.69	30.8%

* Values are shown only for the month in which purchases occurred in the database. Prices of individual items per unit can be found in the link shared in the Availability of Data section.

## DISCUSSION

The data in this study show great variability of prices and extreme price hikes in government-driven purchases related to the COVID-19 response in Ecuador—in particular, during the peak of the pandemic in the country’s initial hotspot of Guayas. Although it is true that greater worldwide demand for medical equipment and supplies drove price increases in both high-income and low- and middle-income countries, government-based contracts in Ecuador diverted from market prices. Furthermore, the national and local governments, public hospitals, and the National Secretariat for Risk Management do not appear to have coordinated bulk negotiations to ensure better prices. In addition, exceptional purchases of ammonium quaternary and hydroxychloroquine by the Ecuadorian government show that decisions were not necessarily based on need or the best available evidence.^25^ As a consequence, government expenditures appear to have been costly and also wasteful.

Irrespective of limits in diagnostic capacity, which are a reality in Ecuador, high prices of RT-qPCR primers and extraction kits in government-driven purchases may have had a negative impact on the number of tests that the Ministry of Health was able to afford.^15^ Accordingly, an increase in the public health budget probably would not have led to greater access to testing through added purchases but instead to further speculation and price hikes. This may help to explain the high price caps set by the government: US$80 per publicly funded and US$120 per privately paid RT-qPCR assay. Unsurprisingly, there was severe undertesting in the country during the study period, with RT-qPCR positivity rates (total confirmed cases as a share of the total number of people tested) between 48% on May 1, 2020^26^ and 42% on July 31, 2020.^27^

Although the current analysis concerns only government-driven purchases, private demand for medical supplies and medicines in a country reliant on imports and with highly concentrated local production of medicines may have also influenced price increases. However, there is no evidence that the government was trying to keep prices down through control mechanisms or at least greater scrutiny and accountability of public purchases. Media reports on corruption in government-driven purchases pointed toward contracts remaining largely unmonitored as the health emergency unfolded,^28,29^ and investing in local production was not a high priority in the government agenda.

Speculative price hikes amid constraints in public spending may have limited patient access to diagnosis and treatment of COVID-19, and protection of both the general population and health workers in clinical settings. Prices of PPE and medicines, including hydroxychloroquine (antimalarial medication that was briefly suggested for COVID-19 treatment) experienced increases in several countries.^30^ Lack of PPE may help to explain why, early in the pandemic (February 2–April 18, 2020), the most impacted occupational sector in Ecuador was health care (19% of total COVID-19 confirmed cases).^31^ Although moderate increases in prices have not been found to negatively influence purchase of PPE, extreme increases such as 1,307% on the price of medical gloves may have had a limiting effect.

In countries such as Brazil, oxygen shortages and price speculation led to the death of vulnerable patients who were not able to procure cylinders that had been reserved for sale to the wealthy.^32^ Similarly, the city of Guayaquil reported shortages and prices up to US$50 for the refill of an oxygen cylinder^23^ in early April, when a record number of people died.

Although the excessive cost of food kits illustrates widespread speculation in COVID-19 related government-driven contracts, it is important to note that nutritional status may have been affected by the number of people who could actually receive this type of aid. Moreover, under such circumstances, requests for people to stay at home or quarantine were difficult to meet when they had to acquire their means of subsistence through informal work or personally procure foodstuffs, further undermining proper prevention and treatment. Direct purchases by the government from farming associations that were already helping in the response could have helped reach more people, together with keeping the economy moving, with the funds that had been allocated for emergency food procurement.^8,9^

Finally, information on COVID-19 vaccine vials is not available through SERCOP or other data sources, and the prices paid for them are unknown at the time of this publication. Unplanned, unethical allocation of doses privileging public officials and their spouses, journalists, and businesspeople has been widely reported,^33^ making it even more relevant than before to correct obscure practices in the use of public health resources.

### Study limitations.

This study has limitations. First, data collection and analysis focused on the most frequently identified products in public contracts citing COVID-19. We do not know what the case is in other, including essential, supplies or equipment. Second, we do not have data on patient access to health care, such as diagnosis and treatment of COVID-19, so we cannot provide conclusions on the impact of price variation. Third, we do not know the factors that may have influenced product pricing, such as global increase in the cost of materials and transportation due to disruptions in supply chains. Nevertheless, the study brings to the fore public purchase practices that may have a detrimental effect on health outcomes.

## CONCLUSION

The COVID-19 pandemic has exposed flaws in health system governance around the world, highlighting the potential for price speculation and unjustified hikes in prices to undermine the effectiveness of country responses. At the beginning of the pandemic, Ecuador suffered one of the most aggressive outbreaks of COVID-19 worldwide, and many questions have remained unanswered regarding the extremely high spike in confirmed cases and excessive deaths in provinces such as Guayas. An unfulfilled need for continuous access to face masks for the general population, as well as food supplies, may have played a role in the increased transmission in the country. Supplies allocation during the COVID-19 response depended on budgetary constraints due to Ecuador’s ongoing financial crisis and consequently required effective use of available funds. Furthermore, additional dependance on out-of-pocket payments warranted price caps and other forms of regulation, as well as greater scrutiny of public purchases.

Improvements in the health system and pandemic preparedness efforts should focus on ensuring adequate investment of public resources and planning for availability of supplies for the prevention of infectious diseases. Anticipating bulk purchases across hospitals, at the least, and across other institutions and sectors, should be prioritized to help guarantee affordable prices not just within the public health sector but also for patients who may be forced to pay out of pocket or use their private insurance. Especially in the case of an inexpensive but apparently highly effective measure such as mandatory or voluntary mask wearing, basic price controls may induce better compliance with self-care activities. Investing in local production of medical supplies and complementary but essential resources such as food should also be considered.
